# The Prevalence and Associated Risk Factors of Fear of Fall in the Elderly: A Hospital-Based, Cross-Sectional Study

**DOI:** 10.7759/cureus.23479

**Published:** 2022-03-25

**Authors:** Minakshi Dhar, Nidhi Kaeley, Prakash Mahala, Vartika Saxena, Monika Pathania

**Affiliations:** 1 Internal Medicine, All India Institute of Medical Sciences, Rishikesh, Rishikesh, IND; 2 Emergency Medicine, All India Institute of Medical Sciences, Rishikesh, Rishikesh, IND; 3 Family and Community Medicine, All India Institute of Medical Sciences, Rishikesh, Rishikesh, IND

**Keywords:** quality of life, activities of daily living, hindi mental state examination, geriatric population, fear of fall

## Abstract

Background

Falls are one of the most common but serious health issues faced by the elderly population. Falls-related injuries not only add to the morbidity and economic burden they also have a significant negative impact on psychological health and the quality of life of the elderly. In this study, we assess the prevalence of fear of falling among the geriatric population and the risk factors associated with fear of falling.

Methodology

This prospective, cross-sectional study was conducted among geriatric patients aged more than 60 years attending the medical outpatient department of a tertiary care hospital in Uttarakhand, India. This study aimed to determine the prevalence of fear of falling among the geriatric population and compare various demographic and clinical parameters in elderly patients with and without fear of falling. Fear of fall was assessed using a questionnaire by asking the elderly if they had fear of falling, how often they had fear of falling, and under which circumstances did they have fear of falling. Information regarding demographics, history of daily activities, and chronic diseases was collected and assessed from all geriatric patients with and without fear of falls.

Results

Almost half (334; 42%) of the geriatric population had a history of fear of falling. Demographic parameters such as age over 80 years, female gender, living alone, and rural background were significantly associated with fear of falling. Similarly, a history of chronic diseases such as stroke, hypertension, and history of visual and motility impairment was significantly related to fear of falls among geriatric patients. Around 70% (571) of geriatric patients aged over 60 years had associated health problems.

Conclusions

This study reinstates that fear of falls is a significant health issue in the elderly age group. It also highlights multiple risk factors related to fear of falling which if controlled can significantly improve the quality of life of the geriatric population.

## Introduction

According to the 2011 census, India is one of the most populous countries in the world, with a population of around 1.21 billion people. People aged 60 and above account for 8.6% of the total population [1,2]. Aging is a dynamic process involving progressive physiological changes associated with functional, morphological, biochemical, and psychological complications. All these changes make the elderly vulnerable to unique problems. Among the elderly, falls are a major health issue, which can be recurrent and multifactorial [3,4]. Annually, accidental falls account for nearly one-third of those aged over 60 years, with 10% of these falls accounting for grievous injuries [5].

Falls can lead to disability, hospitalizations, and early death [6]. Moreover, falls are associated with reduced levels of independence, higher levels of anxiety, and reduced quality of life [7]. It has been seen that fear of falling is not only a foremost health problem amid the elderly who have fallen but also among those who have never experienced a fall. Further, fear of falling can have a devastating impact on the ability to perform activities of daily living, frequency of hospitalizations, and quality of life (QOL) [8-11].

It has been observed that fear of falling instills an attitude of avoidance of physical activity and social isolation leading to deterioration in the physical and mental health of the elderly population. Previous studies have reported variable prevalence of fear of falling ranging from 3% to 85% [12,13]. Fear of falling also contributes to reduced lower limb strength due to ailments such as osteoarthritis and neuropathies. This can result in poor postural control and an increased frequency of falls [14].

There are limited studies, especially in developing countries such as India, assessing the factors and determinants of fear of falling in the elderly population in the hilly region of Uttarakhand, India. This study aims to highlight the prevalence and determinants of fear of falling in the elderly Indian population attending a tertiary care hospital. This will help plan measures to prevent falls in the elderly population and improve their physical and mental well-being.

## Materials and methods

This cross-sectional study was conducted in a tertiary care hospital in north India, which is the biggest and busiest hospital in the region and caters to the hilly population of Uttarakhand and the eastern and western parts of Uttar Pradesh. The research protocol was approved by the Institutional Ethics Committee of All India Institute of Medical Sciences (AIIMS), Rishikesh, India (AIIMS/IEC/17/165). The study population included all elderly people above 60 years of age attending the outpatient geriatric clinic of the medicine department. The prevalence of fear of fall from a previous study was considered to be 25%, the confidence limit was considered to be 5%, and the design effect as the minimum sample size was calculated to be 271. However, we extended the study to six months, and a total of 795 patients were enrolled in the study. The inclusion criteria were elderly people aged 60 and above who had an activity of daily living (ADL) score of 6/6 [14]. People accompanying other patients were included as study participants if their age was 60 years or more. Written informed consent was taken from all participants. Wheelchair and bed-bound patients were excluded from the study. Elderly people unwilling to participate in the study and with terminal illnesses were also excluded. A systematic random sampling method was used to obtain the study sample.

A standardized, semi-structured questionnaire was used for data collection, and an expert physician performed a physical examination. A personal interview was performed for all participants in a separate room adjacent to the outpatient department. The questionnaire collected information on sociodemographic characteristics, questions concerning falls, instrumental activities (shopping, use of telephone, finance management, etc.), use of walking aids, insomnia, and reported morbidity. Detailed history related to comorbidities such as hypertension, diabetes mellitus, arthritis, stroke, and medication was obtained from all geriatric patients and tabulated. The cognitive function of the patients was assessed using the Hindi Mental State Examination (HMSE) questionnaire [15,16]. The HMSE, a test dealing with people who are illiterate, is expected only to screen individuals for cognitive impairment. A tool developed by the Indo-US Cross-National Dementia Epidemiology Study, it comprises 22 items that examine several cognitive capacities (e.g., orientation to time, place, and recognition of objects; memory; attention; language function; concentration; comprehension and expressive speech; motor functioning; and praxis). The total score if all elements are answered is 30 similar to the Mini-Mental State Examination [17]. A history of falls was appraised with a single item: how recurrently have you fallen in the past six months? A wall-mounted stadiometer was used for measuring height. Bodyweight was measured using a standard weighing machine without shoes, and the body mass index (BMI) was calculated using the formula weight (kg)/height^2^ (m). The fear of falling was objectively assessed using the Hindi-translated version of the Fall Efficacy Scale-I (FES-I) [18]. The FES-I is a 16-item questionnaire with excellent psychometric properties with very high internal reliability, which have been determined both in English and in a cross-cultural perspective. Depression was appraised by the Hindi version of the Geriatric Depression Scale (short form) which consists of 15 questions [19]. A score of more than and equal to five suggests depression. History related to discomfort in the neighborhood was also asked. Further, history related to discomfort at home and in the community was probed. The answers yes (one point) and no (zero point) discomfort were recorded. Discomfort at home was perceived as discomfort in the bathroom, kitchen, bedroom, stairs, and when they went out. Community discomfort was related to discomfort at hospitals, parks, market areas, banks, and public spaces. Social support was evaluated by inquiring about accessibility to immediate close relatives and children for help. Yes stood for discomfort present whereas no stood for no discomfort present.

Data were added in a Microsoft Excel spreadsheet and analyzed using SPSS version 21.0 (IBM Corp., Armonk, NY). Mean and standard deviation (SD) were computed for quantitative data. For qualitative data, proportions were computed. Suitable tests of significance such as the chi-square test and t-test were used. Logistic regression was performed to test the association of fear of falling with other variables such as age and gender. All statistical tests were two-tailed, and a p-value of <0.05 indicated statistical significance.

## Results

The study sample consisted of 795 elderly patients above the age of 60 years who were willing to participate in the study. Geriatric patients with a terminal illness and unwilling to participate in the study were excluded from the study. Out of the 795 geriatric patients, 334 (42%) had a history of fear of fall (FES-8-28). In total, 222 (66.4%) patients with a history of fear of falls were females and 112 (33.5%) were males. Around 50% of geriatric patients with a history of fear of falls were more than 80 years of age (Table 1).

**Table 1:** Variables of the geriatric population with and without a history of falls (n = 795). FES: Fall Efficacy Scale

Variables	Number (%)
History of falls in the past six months	284 (35.7)
Falls with injury	73 (25.7)
Fear of falls (FES-8-28)	334 (42)
No fear of falls (FES < 7)	461 (57.9)
Depression (score <5)	86 (10.8)
Associated health problem	571 (71.8)

Overall, 284 (35.7%) geriatric patients had a history of falls, of whom one-fourth of the patients had a history of falls with injury. Approximately 10% of the geriatric patients had a history of depression. The association of fear of falling with demographic parameters is shown in Table 2.

**Table 2:** Comparison of demographic characteristics of the geriatric population with and without a history of falls.

Variable	With a fear of falls (334; 42%)	Without a fear of falls (461; 57.9%)	Chi-square	P-value
Age (years)			13.9	0.001
60–69	222 (38.9)	361 (61.9)		
70–79	75 (52.1)	69 (47.9)		
>80	37 (54.4)	31 (45.6)		
Gender			0.56	>0.05
Males	112 (33.5%)	293 (63.5%)		
Females	222 (66.4%)	168 (36.4%)		
Socioeconomic status			1.13	>0.05
Upper high and higher	114 (34.1)	113 (24.5)		
Middle and lower	220 (65.8)	348 (75.4)		
Educational status			36.35	<0.001
Illiterate	198 (59.8)	174 (37.7)		
Literate	136(40.7)	287 (62.2)		
Comorbidities				
Stroke	53 (15.8)	9 (1.9)	8.42	0.01
Parkinsonism	1 (2.9)	5 (1.0)	0.88	0.40
Hypertension	114 (34.1)	86 (18.6)	9.4	0.001
Sedative drug	30 (8.9)	8 (1.7)	1.25	0.09
Antihypertensive medication	107 (32)	74 (16.05)	28.8	0.000
Antipsychotic medication	5 (1.4)	4 (0.8)	1.86	0.23
Falls			11.58	0.000
Never	153 (45.8)	303 (65.7)		
1	81 (24.2)	116 (25.1)		
>1	100 (29.9)	12 (2.6)		
Medications			18.88	0.001
None	125 (37.4)	211 (45.7)		
1–2 medications	173 (51.7)	194 (42.0)		
>3 medications	36 (10.7)	56 (12.1)		
Marital status				
Living with a spouse (499; 12.7)	173 (51.7)	288 (62.4)	101.6	<0.001
Living without a spouse (296; 37.2)	161 (48.2)	173 (37.5)		
Diabetes mellitus	78 (23.3)	90 (19.5)	8.96	<0.001
Arthritis	155 (46.4)	141 (30.5)	149.32	<0.001
Trouble in vision	152 (45.5)	169 (36.6)	62.6	<0.001
Trouble in hearing	89 (26.6)	86 (18.6)	44.04	<0.001
Discomfort in the neighborhood environment	2.01 ± 1.49	1.85 ± 0.95	6.70	<0.001
Social support	1.99 ± 0.76	2.29 ± 0.87	4.07	<0.001

Fear of falls was statistically significantly (p ≤ 0.05) associated with age. More than 50% of geriatric patients with a history of fear of falling were aged more than 80 years. The educational and marital status was also significantly associated with fear of falling in the geriatric population. Overall, 198 (59.8%) patients with a history of fear of falls were illiterate. Moreover, 161 (48.2%) elderly patients with a history of fear of falls were living without a spouse. Demographic parameters such as gender and socioeconomic status were not significantly associated with fear of falling. There was no significant difference in the history of fear of falls in patients with upper, middle, and lower socioeconomic status. Fear of falling was significantly associated with stroke (53; 15.8%), hypertension (114; 34.1%), diabetes mellitus (78; 23.3%), and arthritis (155; 46.4%). In total, 107 (32%) patients on antihypertensive medication had fear of falling. Moreover, 100 (29.9%) patients with fear of falling compared to 74 (16.05%) patients without a history of fear of falling had a history of falls more than once. Trouble in vision (152; 45.5%) and impaired hearing (89; 26.6%) were also significantly associated with fear of falls. A history of Parkinsonism, sedative drugs, and antipsychotic medication use was not significantly related to a history of fear of falling.

## Discussion

The prevalence of fear of falls among the elderly population in our study was 42%, which is comparable to the study by Kumar et al. and an Indian study by D'souza et al. [7,19]. In our study, 181 (54.1%) patients with fear of falling had a history of falling once or more than once. Similarly, in another study, 45.7% of the subjects with fear of falling had a history of falling. The correlation between fear of falls and frequency of falls has been well established by previous studies [20,21]. The incidence of falls inculcates a psychological fear of falling in the elderly limiting their movement and physical activity. In this study, around 50% of patients with a history of fear of falling were over 80 years of age. This result was consistent with a previous study by Martin et al. [22].

The majority of the elderly experience falls due to unsafe bathrooms, slippery staircases, insufficient lighting, and handrails on the stairs. They have fear of falling both outside and inside their homes. Accessibility to basic needs shops, hospitals, parks, and banks can limit the chances of falling and positively affect the fear of falling in the elderly. In addition to environmental factors, social support and frequent contact with close relatives can limit the fear of falls in the geriatric population significantly leading to the improved mental health of this subset of the population. The elderly should be encouraged to participate in various social and religious activities. It has been observed that the geriatric population is often abandoned by caregivers, rendering them vulnerable to fear of falls. Fear of falls in the elderly leads to restriction of physical activities which impacts their physical as well as mental health [23]. In this study, a significant association was noted between educational status and fear of falling.

Kempen et al. also revealed significant correlation between fear of falls and female sex, restricted physical activity, previous history of falls, and educational status as education has an overall impact on the growth and development of all individuals. It influences the economic status and the ability to take good care of themselves, both physically and medically, among the geriatric population. Education status is associated with fear of falls in the elderly [24]. In our study, gender was significantly associated with fear of falling. Previous studies have shown higher prevalence of fear of falling among elderly females. The underlying reason for this could be that females have a weaker musculoskeletal system and have a higher tendency to develop osteoporosis. The fear of falling in the elderly discourages them to perform physical activities. This leads to deterioration in their muscle strength and postural control. Pain has also been highlighted as one of the contributory factors affecting fear of falls [25,26].

In this study, a history of antipsychotic medication and hypnotics did not significantly correlate with a history of fear of falls. The use of anxiolytics and hypnotics is very common in the elderly population due to ailments such as depression, anxiety, and lack of sleep. These drugs not only decrease alertness in the geriatric population but also affect their postural control. Several studies have investigated the effect of polypharmacy on fear of falls in the elderly population [27-29]. Hence, more studies are needed to establish this correlation. In this study, a history of falls has been significantly related to the fear of falls in the geriatric population. Falls in the elderly lead to fractures and increased risk of hospitalization which further enhance fear of falls in them. Consequently, they limit their physical activity. This lack of physical activity increases the risk of developing metabolic diseases, such as obesity, coronary syndrome, diabetes mellitus, stroke, and hypertension in the geriatric population. Previous studies have highlighted this positive correlation between fear of falling and a history of comorbidities, such as hypertension, stroke, and diabetes mellitus [27].

Aging leads to physical, psychological, and systematic metabolic derangement, and the elderly are more prone to miscellaneous non-communicable diseases, such as osteoarthritis, diabetic mellitus with microangiopathies, such as diabetic neuropathy and cardiovascular diseases [27]. Consequently, they are on multiple drugs, rendering them prone to falls as well the fear of falling [28]. It is prudent to encourage the elderly for regular follow-up outpatient department and emergency visits to note the side effects of these drugs and other vital parameters [27]. Aging is associated with lower functional reserve as well as low self-esteem. In addition, harsh and unfriendly environmental conditions as well as lack of social support can significantly affect fear of falls in the elderly population. A similar observation has been made by Tiwari et al [16].

The first encounter of the elderly population is with family and emergency physicians. It is very important for them to understand the basic needs of the elderly population. Fear of falls is one of the important aspects of their management. Most of the risk factors discussed are modifiable, such as improving external environment, education status, improving awareness, providing them timely medical aid to prevent the development of comorbidities, as well as encouraging them for regular follow-up medical check-ups. It is the need of the hour for society as well as family physicians to understand the factors affecting fear of falls in the elderly and take corrective measures.

## Conclusions

Fear of falls is significantly affected by parameters such as illiteracy, age, comorbidities such as stroke and hypertension, polypharmacy, and previous history of falls. Most of these factors are modifiable risk factors, with one affecting the other. Thus, the early identification of these factors can lead to significant improvement in their quality of life by not only preventing falls but also eliminating the fear of falls. A multidirectional approach should be considered while addressing a history of falls and fear of falls in the geriatric population.
